# Training, Awareness, and Clinical Perspectives of Pediatric Dentists on Headache and Migraine Management: A National Survey Study

**DOI:** 10.3390/children12080968

**Published:** 2025-07-23

**Authors:** Samantha Glover, Linda Sangalli, Caroline M. Sawicki

**Affiliations:** 1 Department of Pediatric Dentistry and Dental Public Health, University of North Carolina, Chapel Hill, NC 27599, USA; sag40@email.unc.edu; 2 College of Dental Medicine—Illinois, Midwestern University, Downers Grove, IL 60515, USA; lsanga@midwestern.edu

**Keywords:** headache and migraine management, healthcare collaboration, provider knowledge, chronic pain in children, orofacial pain, pediatric dentistry, pediatric headache disorders, oral–systemic health

## Abstract

**Background/Objectives:** Migraine affects approximately 3–10% of school-aged children and up to 28% of adolescents, with prevalence increasing during adolescence. For pediatric specialty providers, increased awareness of this condition may influence patient care. This study examined pediatric dentists’ education, clinical exposure, and perceived knowledge gaps related to pediatric migraine, with the goal of identifying barriers to recognition and referral, as well as informing future training to support accurate diagnosis and interdisciplinary care. **Methods:** A 28-item electronic questionnaire was distributed to all members of the American Academy of Pediatric Dentistry, including pediatric dentists and postgraduate pediatric dental residents, assessing knowledge, beliefs, clinical experience, and interest in further training regarding pediatric headache/migraine management. Respondents with and without previous training were compared in terms of general understanding using *t*-tests; a linear regression model analyzed predictors of provider awareness regarding links between oral conditions and headache/migraine. **Results:** Among 315 respondents, the mean self-perceived awareness score was 2.7 ± 1.3 (on a 0–5 scale). The most frequently identified contributing factors were clenching (73.7%), bruxism (72.4%), and temporomandibular disorders (65.7%). Nearly all respondents (95.2%) reported no formal education on headache/migraine prevention, yet 78.1% agreed on the importance of understanding the relationship between oral health and headache/migraine. Respondents with prior training were significantly more aware (*p* < 0.001) than those without prior training. Educating families (*p* < 0.001), frequency of patient encounters with headache (*p* = 0.032), coordination with healthcare providers (*p* = 0.002), and access to appropriate management resources (*p* < 0.001) were significant predictors of providers’ awareness. **Conclusions:** Pediatric dental providers expressed strong interest in enhancing their knowledge of headache/migraine management, highlighting the value of integrating headache/migraine-related education into training programs and promoting greater interdisciplinary collaboration.

## 1. Introduction

Migraine is one of the most common types of primary headache and ranks as the third-most prevalent disorder globally, posing a significant and disabling public health burden [1,2]. The International Classification of Headache Disorders 3rd edition (ICHD-3) categorizes migraine into two major subtypes, migraine with and without an aura, and defines diagnostic criteria based on frequency and duration, with attacks lasting 4–72 h and occurring on at least five separate occasions [3]. In pediatric populations, migraine is a leading causes of emergency department visits for recurrent headache, contributing to increased healthcare utilization, school absenteeism, and reduced quality of life [4]. However, diagnosis is often delayed and management complicated by limited provider awareness, distinct signs and symptoms compared to migraine manifestation in adults, and inadequate access to pediatric headache specialists [5]. In children and adolescents, diagnosis relies heavily on clinical history and symptom patterning, which may complicate recognition and prolong time to treatment initiation [6]. Although multidisciplinary approaches have shown promise, the literature remains scarce on management approaches in interdisciplinary or primary care settings [7].

Given that headaches (and particularly migraine) are a common comorbidity among patients with temporomandibular disorders (TMDs), and that both conditions share overlapping biological and psychosocial risk factors, there is a clear opportunity for greater interdisciplinary collaboration amongst pediatric oral healthcare providers [8,9]. Large-scale studies, including the OPPERA (Orofacial Pain: Prospective Evaluation and Risk Assessment) cohort, have shown that individuals with TMD are at significantly increased risk for developing migraine, likely due to shared mechanisms such as central sensitization, impaired pain modulation, and psychosocial stress [10,11,12,13]. These findings highlight the importance of early recognition and coordinated care strategies. Pediatric migraine may have lasting consequences that extend into adulthood, including physical, emotional and psychosocial effects [5,14]. Furthermore, symptom overlap between migraine and neurovascular orofacial pain (e.g., photophobia and phonophobia) can complicate diagnosis and lead to misattributed pain etiologies [5,15]. As frontline providers for pediatric orofacial pain complaints, pediatric dentists are uniquely positioned to support early recognition of migraine symptoms, especially when facial or jaw pain is the chief complaint, and to facilitate appropriate referral and care coordination. Although precise data are lacking, case series have described instances in which migraine was misdiagnosed as dental pain, resulting in unnecessary dental procedures such as extractions [16,17]. Pediatric dentists may support headache/migraine management by screening for hallmark symptoms during routine dental visits, including photophobia, phonophobia, nausea, or pain exacerbated by routine activities. When these features are present, providers can educate families about the potential link to headache disorders and refer the patient to a pediatrician or neurologist for further evaluation. Simple tools, such as symptom checklists or referral algorithms, could help integrate this process into routine care without significantly increasing visit time. While dentists receive training in the differential diagnosis of orofacial pain, formal instruction on the recognition and co-management of primary headache disorders, such as pediatric migraine, is often limited or absent in dental education. As a result, it remains unclear whether pediatric dentists feel their current knowledge is sufficient, or whether there is a need for continued education and refresher training, to recognize migraine-specific symptoms and contribute to interdisciplinary care for affected patients. While the Commission on Dental Accreditation (CODA) standards for pediatric dentistry (USA—2025) outlines essential competencies in the diagnosis and management of oral diseases and craniofacial development in pediatric populations, current curricula offer limited emphasis on primary headache disorders, including migraine, or on interdisciplinary care coordination for orofacial pain conditions that extend beyond the dentition [18].

Increased awareness among pediatric dentists could facilitate earlier referral to neurologists or pain specialists, ultimately improving patient outcomes and reducing the risk of misdiagnosis or unnecessary interventions. Additionally, given the emerging recognition of the relationship between oral parafunctional habits, such as clenching, bruxism, and TMD, and headache disorders, dental providers may be well-positioned to recommend behavioral interventions or deliver occlusal appliance therapies as adjuncts to comprehensive migraine management. However, the extent to which pediatric dental providers currently engage in headache/migraine-related care, feel equipped to do so, or express interest in expanding their role, remains largely unknown.

This study aimed to evaluate pediatric dentists’ training, clinical experience, and perceived knowledge gaps related to pediatric headache/migraine, with the goal of identifying gaps that may inform future education and interdisciplinary care strategies. We hypothesized that pediatric dentists and residents would report limited formal training and low self-perceived awareness of the oral–systemic connections relevant to pediatric headache/migraine.

## 2. Materials and Methods

### 2.1. Study Design

This cross-sectional survey study was reviewed by the University of North Carolina Institutional Review Board (IRB) and deemed exempt (24-0459, 20 February 2025). An anonymous online questionnaire was distributed via Qualtrics to all members of the American Academy of Pediatric Dentistry (AAPD). Eligible participants needed to be completing or have already completed specialized training in pediatric dentistry at a U.S. CODA-accredited advanced dental education program. Electronic informed consent was obtained from respondents prior to participation. In accordance with local IRB regulations, participants were not obligated to respond to all survey items.

### 2.2. Survey Assessment Tool

The anonymous survey (Supplementary Materials) was co-developed by a pediatric dentist (C.M.S.), a pediatric dentistry resident (S.G.), and an orofacial pain specialist (L.S.), with expertise in pediatric dentistry (C.M.S., S.G.) and pediatric orofacial pain (L.S.). Survey content was also reviewed by external content experts to ensure relevance and appropriateness of questions, as well as readability and accessibility. The finalized survey was also reviewed by the Odum Institute for Research in Social Science at the University of North Carolina to evaluate survey structure, item clarity, and formatting. While content review was conducted by subject matter experts, the instrument has not yet undergone formal psychometric validation. While the term “headache/migraine” does not appear as such in the International Classification of Headache Disorders (ICHD-3), this phrasing has been used to be inclusive of different forms of headache and migraine. This wording enables a more relevant clinical application for the population of our study. The final survey consisted of 28 items across 5 sections. Responses to all sections were optional. Section 1 assessed respondents’ professional background, years of experience, additional training on pediatric headaches/migraine, location of work and primary practice setting. Section 2 evaluated general understanding and provider’s knowledge into an oral health and headache/migraine link, as well as experience in educating families in practice. Section 3 involved questions on patient identification and practice interventions, inquiring on frequency of identified patients as well as frequency of counseling and working with an interdisciplinary pediatric headache team. Section 4 included questions on pediatric dentists’ opinion on their role in headache/migraine management as well as future focused questions on implementation into practice. The final section of this survey assessed sociodemographic characteristics. Opportunities to elaborate on close-ended responses were provided throughout the process via optional open text boxes.

### 2.3. Data Analysis

Descriptive statistics were used to summarize respondents’ demographic, professional characteristics, and study variables. Differences in providers’ general understanding of the link between oral conditions and headache/migraine were examined across years of professional experience using a one-way analysis of variance (ANOVA), with Bonferroni as post hoc test for comparison.

Next, subgroup analyses were computed. Independent *t*-tests compared respondents with and without prior dedicated training in headache/migraine prevention, access to adequate resources, and engagement in related behaviors (e.g., educating patients’ families) in terms of their general understanding. Chi-square tests were used to investigate whether frequency of encountering patients reporting headaches/migraine differed based on years of professional experience, family education behaviors, opportunities for interprofessional coordination, and approaches to managing pediatric headache/migraine. Effect sizes were reported using Cohen’s *d* for *t*-tests, Cramer’s V for chi-square tests, and eta-squared (η^2^) for ANOVAs. According to conventional thresholds, an effect size of 0.2 is considered small, 0.5 medium, and 0.8 large [19].

Finally, a multiple linear regression model was conducted to identify predictors of providers’ awareness of the link between oral conditions and pediatric headache/migraine.

All the analyses were conducted with SPSS (IBM SPSS Statistics Macintosh, Version 29.00, IBM Corp., Armonk, NY, USA), setting α at <0.05.

## 3. Results

### 3.1. Participants

**Figure 1:**
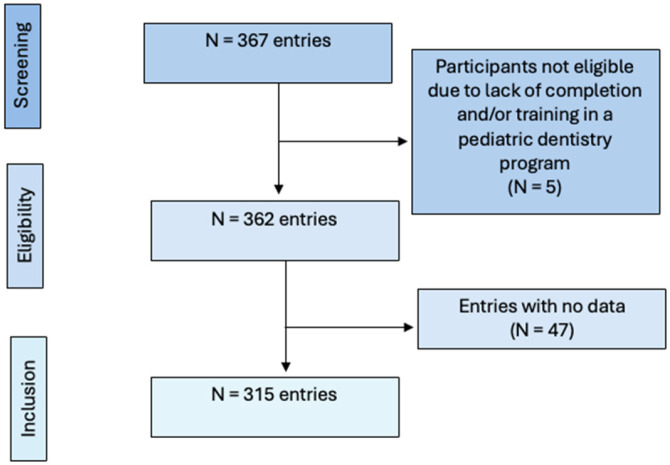
Flowchart of study participants.

Out of 367 total responses, 5 (1.4%) derived from participants who did not complete or were not currently completing a specialized training in pediatric dentistry, while 47 (12.8%) were submitted without any data. Thus, these entries were excluded from the final analysis, leaving a total of 315 participants (Figure 1). Based on 315 completed surveys out of approximately 6500 reachable AAPD members, the response rate was 4.8%.

Most of the respondents (81.6%) were practicing pediatric dentists primarily working in private practice (66.0%) and academia (21.3%), while 15.9% of the responses derived from pediatric dental residents. Most of the participants indicated working in suburban (53.0%) and urban areas (36.5%). Almost half of the respondents (46.3%) had over 20 years of experience. Demographic characteristics of the total sample are presented in Table 1.

### 3.2. Providers’ General Understanding

**Figure 2:**
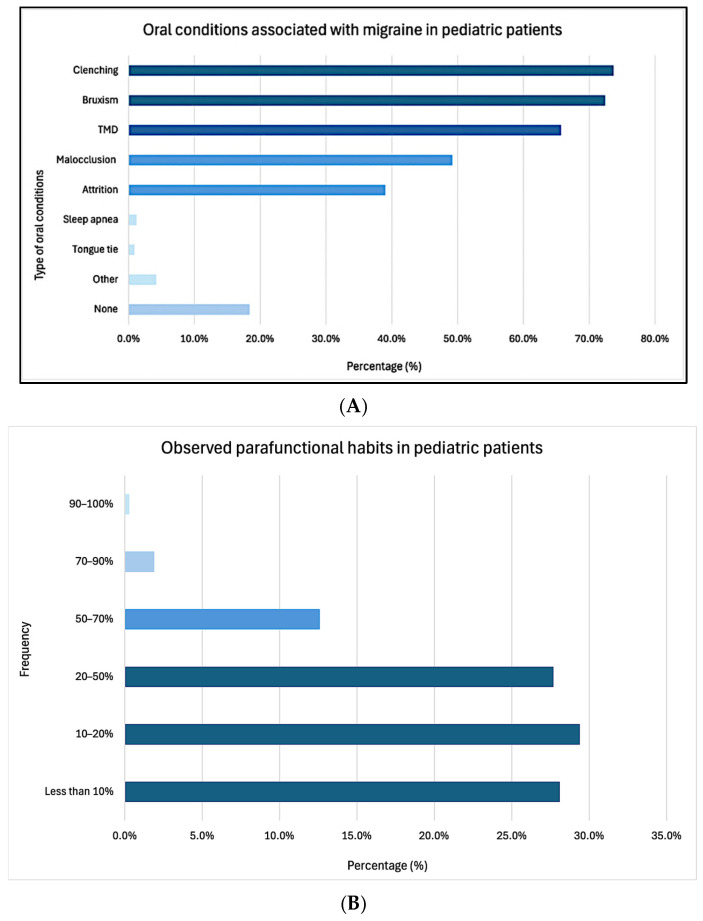
Type of oral conditions perceived to be associated with headache/migraine in pediatric patients (**A**), frequency of observed parafunctional habits (**B**).

On average, respondents reported a self-perceived awareness score of 2.7 ± 1.3 (on a 0–5 numerical rating scale) regarding the potential contribution of oral conditions to headache/migraine in pediatric patients. The most frequently identified contributing factors were clenching (73.7%), bruxism (72.4%), and TMD (65.7%), among others (Figure 2A). A majority of respondents (85.2%) estimated that less than 50% of their pediatric patients exhibited parafunctional habits such as teeth grinding or clenching (Figure 2B).

**Figure 3:**
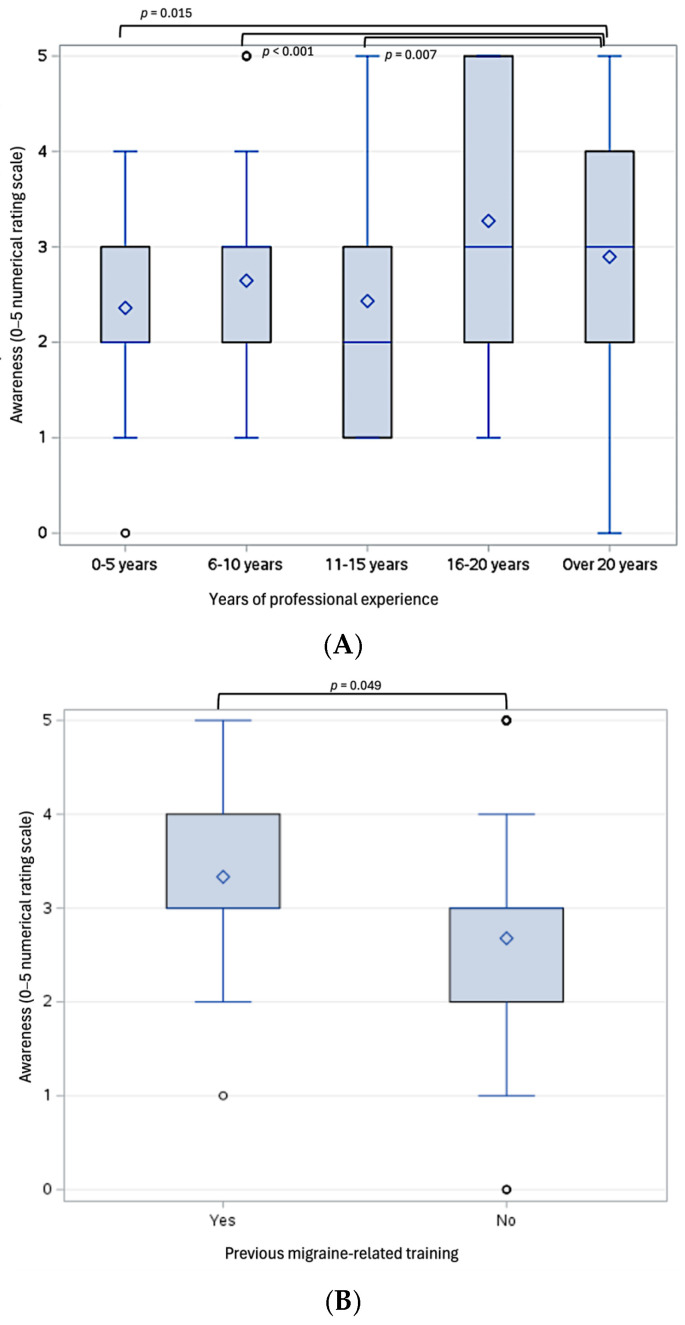

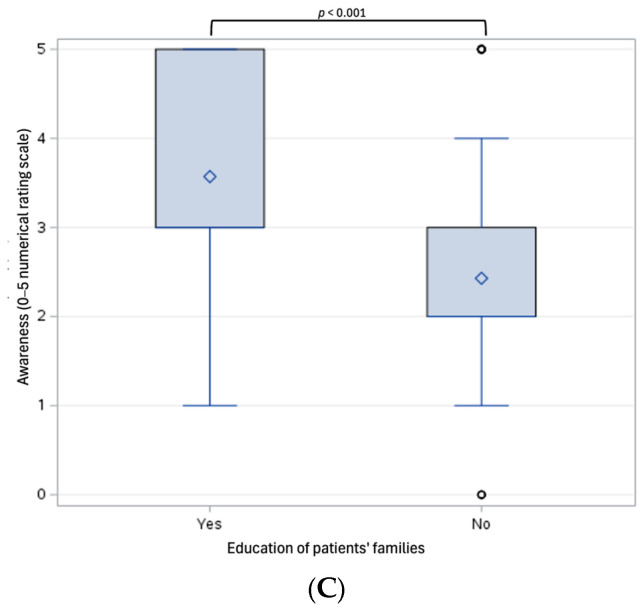
Box plots illustrating statistically significant differences in pediatric dentists’ awareness of the link between oral conditions and pediatric headache/migraine (measured on a 5-point Likert scale), based on years of professional experience (**A**), previous headache/migraine-related training (**B**), and behaviors related to educating patients’ families (**C**). ♢ represents the mean; **○** represents outliers, which fall outside 1.5 times the interquartile range (IRQ) from the lower or upper quartiles.

Pediatric dentists with over 20 years of experience reported significantly higher awareness of this connection (4.5 ± 0.9) compared to those with 0–5 years (3.3 ± 1.7, *p* = 0.015), 6–10 years (2.9 ± 1.8, *p* < 0.001), and 11–15 years of experience (3.3 ± 1.8, *p* = 0.007, Figure 3A). These differences in awareness occurred despite no significant differences in reported training on the role of pediatric dentists in migraine prevention or management during their education (X^2^(4) = 1.176, *p* = 0.882).

A large majority (95.2%) indicated that their formal educational background did not include training on the role of pediatric dentists in headache/migraine prevention or management. Only 34.2% felt they were equipped with resources to address the potential oral health–headache/migraine connection. Respondents who reported being equipped with such resources demonstrated significantly higher awareness of their link compared to those who were not (3.4 ± 1.3 vs. 2.4 ± 1.1, *p* < 0.001, Cohen’s *d* = 0.91). Similarly, only 24.4% reported educating families on the relationship between oral conditions and headache/migraine in their pediatric patients. Those who had received headache/migraine-related training (N = 15) and those who reported educating families on the oral condition–headache/migraine connection (N = 77) also demonstrated significantly higher awareness compared to those who did not receive any training (3.3 ± 1.1 vs. 2.7 ± 1.3, *p* = 0.049, Cohen’s *d* = 0.52, Figure 3B) and those who did not educate the families on such a link (3.6 ± 1.2 vs. 2.4 ± 1.1, *p* < 0.001, Cohen’s *d* = 0.99, Figure 3C).

A multiple regression was performed to predict providers’ awareness of the oral conditions-headache/migraine connection based on previous migraine-related training, years of professional experience, interprofessional collaboration, access to management resources, family education behaviors, and frequency of patient encounters involving headaches. The model significantly predicted providers’ awareness (F(6, 303) = 19.873, *p* < 0.001, R^2^ = 0.268). Specifically, educating families (*p* < 0.001), frequency of patient encounters with headache (*p* = 0.032), coordination with other healthcare providers (*p* = 0.002), and access to appropriate management resources (*p* < 0.001) were statistically significant predictors.

### 3.3. Patient Identification and Practice Interventions

Over two-thirds of respondents (71.0%) indicated that their pediatric patients rarely complain about headaches during their consultation or rarely seek advice from their pediatric dentist regarding headaches, without any differences based on type of primary practice setting (X^2^(12) = 13.616, *p* = 0.326, Cramer’s V = 0.12). However, providers with 20 or more years of experience were significantly more likely to encounter pediatric patients who complained about headaches either frequently (2.8%) or occasionally (19.3%) compared to their less experienced counterparts (X^2^(12) = 22.815, *p* = 0.029, Cramer’s V = 0.27). Respondents who reported educating families on the connection between oral conditions and headache/migraine were also more likely to have patients complaining either frequently (5.2% vs. 0.0%) or occasionally (24.7% vs. 10.3%) compared to those who did not provide such education to their patients (X^2^(3) = 25.521, *p* < 0.001, Cramer’s V = 0.29). Those providers also reported significantly more patients who actively sought advice about headaches during consultations, either frequently (5.2% vs. 0.4%) or occasionally (20.8% vs. 3.9%, X^2^(3) = 47.088, *p* < 0.001, Cramer’s V = 0.39). Moreover, providers who encountered pediatric patients complaining of headache were more likely to coordinate care with other healthcare professionals, such as pediatricians, neurologists, and orofacial pain specialists (X^2^(3) = 29.111, *p* < 0.001, Cramer’s V = 0.31). They were also more likely to report being equipped with resources to address the oral health–headache/migraine connection (X^2^(3) = 10.339, *p* = 0.016, Cramer’s V = 0.18) and to actively collaborate in managing pediatric migraine (X^2^(6) = 25.833, *p* < 0.001, Cramer’s V = 0.30). Similarly, respondents whose patients frequently (3.5% vs. 0.0%) or occasionally (16.0% vs. 1.2%) sought advice about headache were more likely to coordinate interprofessional care (X^2^(3) = 59.774, *p* < 0.001) and to be involved in headache/migraine management (X^2^(6) = 49.873, *p* < 0.001, Cramer’s V = 0.41).

**Figure 4:**
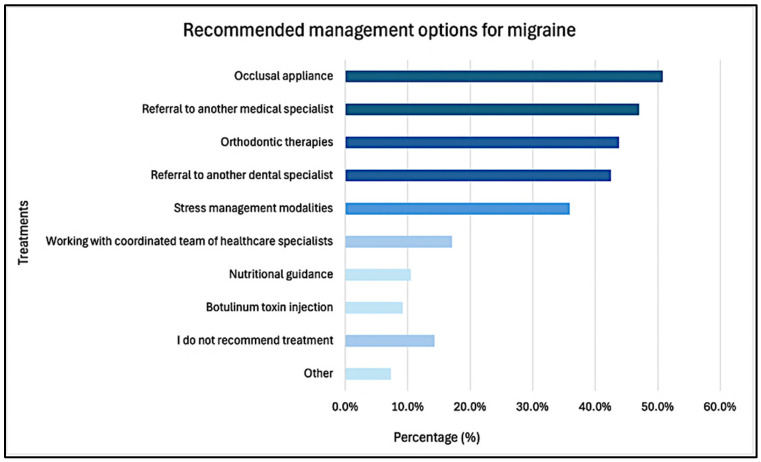
Recommended treatment management for migraine in pediatric dental patients.

The most common recommended approaches for headache/migraine management included nighttime occlusal appliances (50.8%), followed by referral to medical (47.0%) and dental (42.5%) specialists, orthodontic therapies (43.8%), and stress management strategies (35.9%, Figure 4).

### 3.4. Provider Opinion and Future Focus

As high as 78.1% agreed—either somewhat (45.2%) or strongly (32.9%)—that it is important for pediatric dentists to understand the oral health–headache/migraine relationship.

**Figure 5:**
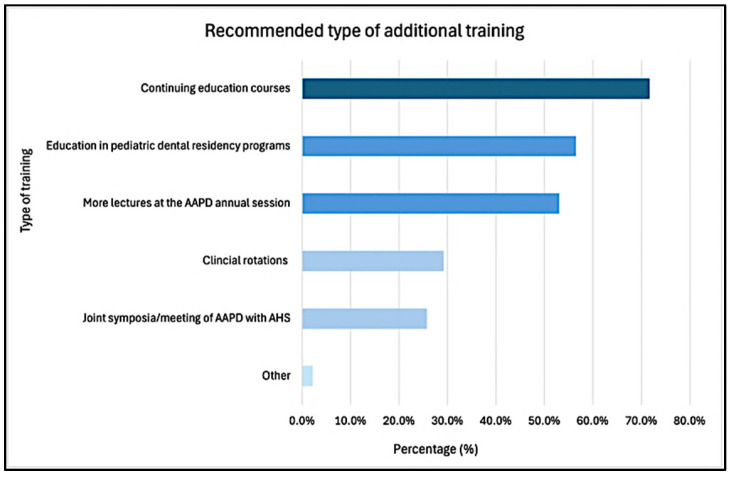
Recommended resources of additional training for pediatric headache/migraine management. AAPD: American Academy of Pediatric Dentistry; AHS: American Headache Society.

Additionally, 85.5% of respondents endorsed the need for increased interdisciplinary collaboration in the management of pediatric headache/migraine. Similarly, 83.2% expressed the need for further training and research in this area. Preferred modalities included continuing education courses (71.7%), integration into pediatric dental residency curricula (56.5%), and more lectures at the AAPD annual session, among others (Figure 5).

**Figure 6:**
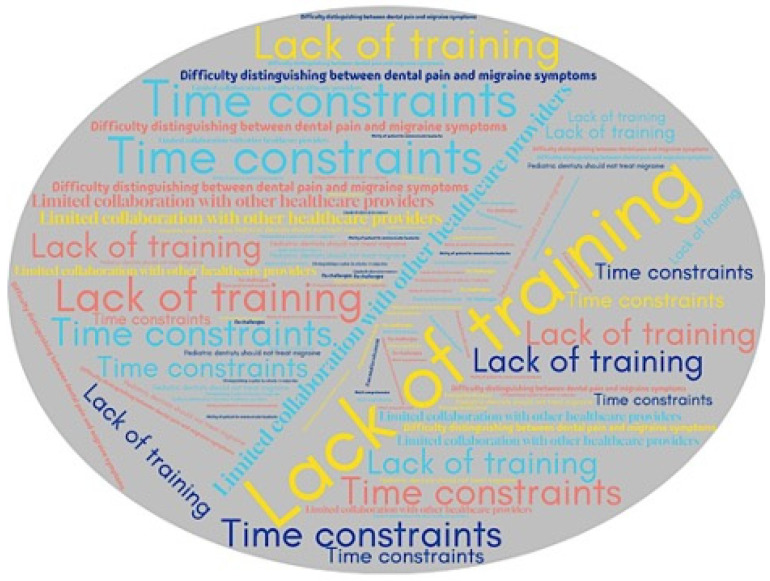
Word cloud illustrating the reported challenges in implementing headache/migraine management into pediatric dentistry practice. Larger size and more frequently repeated items reflect challenges more commonly mentioned by respondents.

The most frequently reported barriers to implementing headache/migraine management into practice were lack of training of formal training or education on the topic (73.7%), limited collaboration with other healthcare providers (36.5%), challenges in distinguishing between dental pain and headache/migraine symptoms (30.8%), and time constraints (28.9%). Only 2.1% of respondents believed that pediatric dentists should not be involved in headache/migraine management in their pediatric patients (Figure 6).

## 4. Discussion

Despite headaches and, specifically migraine, having reciprocal linkages with clinical findings in the pediatric dental setting, this topic remains underemphasized in current training programs and continuing education. The findings of this survey support our initial hypothesis that pediatric dentists and residents report limited formal training and low self-perceived awareness of the oral–systemic connections relevant to pediatric headache/migraine. Respondents’ mean awareness score of 2.7 ± 1.3 (on a 0–5 numerical rating scale) regarding the contribution of oral conditions to headache/migraine highlights an area of opportunity for further education in this area. While the study did not evaluate clinical outcomes, the reported lack of formal training and limited awareness among providers suggest opportunities for ongoing education to strengthen interdisciplinary care for pediatric patients experiencing headache/migraine. Although the AAPD acknowledges primary headaches as common in childhood and adolescence and describes migraine among potential sources of orofacial pain, current guidelines do not include diagnostic criteria for screening and recognition or co-management strategies for migraine. It is important to note that pediatric dentists are not expected to diagnose migraine, but rather to recognize when a child’s symptoms may be consistent with migraine or other primary headache disorders, and to facilitate appropriate referral to a medical provider for diagnosis and management. However, such content is largely absent from pediatric dental curricula and training programs [18,20,21].

It is possible that clinical experience and time in the field contribute to greater awareness, as pediatric dentists with over 20 years of experience reported a significantly higher awareness of a connection between oral health and headache/migraine. This is notable, as these differences in awareness occurred despite no reported differences in training on the role of pediatric dentists in headache/migraine prevention or management during their education. In fact, only 34.2% of respondents felt they were equipped with the resources to address the potential oral health–headache/migraine connection. Additionally, the most frequently identified contributing factors included clenching, bruxism, and TMD. It is also important to note that while the survey focused on migraine, some reported associations, such as those involving parafunctional habits or TMD, may reflect broader provider beliefs about headache etiology rather than migraine specifically. While some literature supports associations between these factors and pediatric headache, research on this link remains limited and methodologically challenging to study in pediatric populations [22]. Effective pediatric care extends beyond treating oral health in isolation but understanding the child in a broader context of their emotional, psychosocial, and overall well-being.

The need for interdisciplinary care and education resources in this realm extends beyond dentistry. The American Headache Society, for example, has developed the “First Contact-Headache in Primary Care” program to provide healthcare providers with the tools to improve headache and migraine care access [23]. The importance of working with interdisciplinary teams cannot be overstated as the ability to coordinate with and refer to other healthcare professionals, such as pediatricians, neurologists, orofacial pain specialists, may be essential for patients debilitated by migraine. Notably, 85.5% of respondents endorsed the need for enhanced interdisciplinary collaboration in pediatric headache/migraine management. Multifaceted, team-based approaches have shown effectiveness in addressing this condition by targeting its intricate etiologies [7].

While headache/migraine management may not be a component of formal education within pediatric dentistry, the findings of this study highlight a call to action to proactively educate early-career providers in this field. This need is further supported by the finding that respondents who felt equipped with educational resources and understanding demonstrated significantly greater awareness of the potential connection between oral health conditions with migraine in pediatric populations. Among respondents, the greatest barrier to implementing headache/migraine management into practice was lack of formal training or education on the topic. Similar gaps in training have also been reported internationally. For instance, a survey of Brazilian orthodontists found that although many providers encountered patients with migraine symptoms, most felt unprepared to diagnose or manage them due to a lack of formal education in headache-related care [24]. These findings highlight a broader, global need to integrate content on headache disorders and interdisciplinary pain management into dental education and continuing professional development.

The distinction in pathophysiological mechanisms between adult and pediatric migraine, largely due to continuous neural development in children, situates pediatric dentists in a unique role for prompt recognition of orofacial pain symptoms, facilitating both proactive clinical response and timely referrals [25]. Because migraine research and pharmacological interventions have traditionally focused on adult populations, directly extrapolating findings to pediatric populations is often unreliable, and unaddressed migraine symptoms in childhood may carry significant long-term consequences. Non-pharmacological treatments, such as cognitive behavior therapy, have demonstrated efficacy in reducing chronic migraine episodes in children and are often most effective when integrated with pharmacologic approaches [26]. Given that adults with migraines frequently experience comorbid anxiety and depression, early identification and management of pediatric somatic symptoms is critical to mitigating long-term psychological and functional impact [26,27]. For pediatric dental providers, awareness of migraine signs and symptoms is especially important due to their overlap with TMD-related pain and associated functional limitations, as well as the risk of psychological distress that may exacerbate orofacial pain conditions over time [14,28,29].

Open dialogue between providers and families is essential, yet if pediatric dental providers are unaware of the potential connection between oral conditions and headache/migraine, key symptoms may go unrecognized or unreported during visits. In fact, respondents who reported educating families on the connection between oral conditions and headache/migraine were also more likely to have patients complaining either frequently (5.2% vs. 0.0%) or occasionally (24.7% vs. 10.3%) compared to those who did not provide such education. These findings suggest that provider awareness directly influences patient disclosures, reinforcing that education begins with a strong knowledge base and the ability to inquire effectively about relevant symptoms and medical history. When equipped with this knowledge, providers are better positioned to refer, educate, and support families appropriately.

Given the frequency of biannual preventive visits, and potentially more for restorative or behavioral concerns, pediatric dentists often maintain more consistent clinical contact with children than many other providers. With only 2.1% of respondents believing that pediatric dentists should not be involved in headache/migraine management, it is evident that both perceived value and professional interest are high. However, greater emphasis on headache/migraine recognition and referral strategies within pediatric dental training and continuing education may be needed to fully integrate this role into routine practice.

Findings from this study illustrate a knowledge gap not only across different generations of pediatric dentists and residents, but also within educational content and interdisciplinary care models. Similar studies piloting interprofessional healthcare approaches in pediatric medicine and dentistry have shown promise in improving referral pathways and care coordination [30]. Future research could explore the development and evaluation of online training modules or continuing education programs, incorporating feedback from pediatric dentists and residents on content retention and clinical applicability. Additionally, integrating dentistry into headache teams or pediatric headache/migraine clinics may provide a practical framework to foster interdisciplinary collaboration.

The results described above should be interpreted in light of the study’s limitations. The response rate was modest (~4.8%), which limits generalizability and raises the potential for non-response bias. It is likely that individuals with greater interest in headache/migraine or orofacial pain were more inclined to participate. However, similar response rates have been reported in prior studies surveying pediatric dental providers, including those evaluating pain management practices and TMD screening behaviors, suggesting that the rate observed here is consistent with research in this field [31,32]. A second limitation is sampling bias, as responses were collected only from AAPD members and may not be representative of the broader pediatric dental community. Additionally, the voluntary nature of survey participation introduces the potential for non-response bias, whereby individuals with limited interest or knowledge of the topic may have been less likely to respond, thus potentially skewing findings toward participants with stronger engagement or opinions. Finally, the small number of respondents with formal headache/migraine-related training limits subgroup comparisons and highlights the need for additional research with larger and more diverse samples. While our study did not evaluate whether insufficient training leads to diagnostic errors or treatment delays, the findings highlight a widespread interest in additional guidance related to headache/migraine, indicating that pediatric dental providers themselves perceive this as a relevant clinical and educational need

## 5. Conclusions

The findings of this study revealed that the vast majority of respondents had not received formal education related to pediatric headache/migraine and reported low self-perceived awareness of the link between oral health and headache/migraine symptoms. Despite this, most participants believed that pediatric dentists should understand this relationship and expressed strong interest in additional training opportunities and interdisciplinary collaboration. Providers who had received headache/migraine-related training, educated families on the topic, or coordinated care with other professionals were significantly more aware of migraine–oral health connections. These findings highlight a need to improve educational content related to pediatric headache/migraine within dental curricula and continuing education programs, with an emphasis on early recognition, family communication, and referral pathways. Improved access to targeted education in this area may enhance care coordination, reduce misdiagnosis or inappropriate management, and ultimately support more comprehensive, patient-centered care, while also strengthening collaboration across pediatric healthcare teams.

## Figures and Tables

**Table 1:** Demographic characteristics of participants.

Variables	Answer Options	Total (N = 315)
Age	25–29 years old	33 (10.5%)
	30–39 years old	66 (21.0%)
	40–49 years old	59 (18.7%)
	50–59 years old	65 (20.6%)
	60 or older	72 (22.9%)
	Prefer not to answer	2 (0.6%)
	Missing	18 (5.7%)
Sex	Male	133 (42.2%)
	Female	162 (51.4%)
	Prefer not to answer	2 (0.6%)
	Missing	18 (5.7%)
Ethnicity	Hispanic or Latinx	17 (5.4%)
	Not Hispanic or Latinx	255 (81.0%)
	Prefer not to answer	25 (7.9%)
	Missing	18 (5.7%)
Race	White	219 (69.5%)
	Black or African American	16 (5.1%)
	Asian	39 (12.4%)
	Other	6 (1.9%)
	Prefer not to answer	17 (5.4%)
	Missing	18 (5.7%)
Professional role	Retired pediatric dentist	8 (2.5%)
	Practicing pediatric dentist	257 (81.6%)
	Pediatric dental resident	50 (15.9%)
Practice setting	Private practice	208 (66.0%)
	Academia	67 (21.3%)
	Private practice and academia	27 (8.6%)
	Community/public health clinic	11 (3.5%)
	Other	2 (0.6%)
Type of location	Urban	115 (36.5%)
	Suburban	167 (53.0%)
	Rural	33 (10.5%)
Region of practice	Northeast	90 (28.6%)
	Midwest	58 (18.4%)
	West	60 (19.0%)
	Southeast	74 (23.5%)
	Southwest	33 (10.5%)
Years of experience	0–5 years	83 (26.3%)
	6–10 years	34 (10.8%)
	11–15 years	30 (9.5%)
	16–20 years	22 (7.0%)
	Over 20 years	146 (46.3%)

Values are presented as frequencies and percentages.

## Data Availability

The original contributions presented in the study are included in the article/Supplementary Materials; further inquiries can be directed to the corresponding author.

## Supplementary Materials

The following supporting information can be downloaded at https://www.mdpi.com/article/10.3390/children12080968/s1, Survey used in the current study.

## Abbreviations

The following abbreviations are used in this manuscript:

CODA	Commission on Dental Accreditation
ICHD	International Classification of Headache Disorders
TMD	Temporomandibular Disorder
AHS	American Headache Society
AAPD	American Academy of Pediatric Dentistry
CBT	Cognitive Behavioral Therapy
