# Supplementary material for: Training, Awareness, and Clinical Perspectives of Pediatric Dentists on Headache and Migraine Management: A National Survey Study
Source: Children (Basel). 2025 Jul 23;12(8):968. doi: 10.3390/children12080968 (PMC12384611; doi:10.3390/children12080968)
Supplement: Supplementary file 1 [file children-12-00968-s001.zip › children-3734981-supplementary.pdf]

Tools Saving... Draft

Preview Publish

Toothaches or Headaches? Pediatric Dentists’ Management of Migraines

ExpertReview score Great

Study Information Cover Sheet

Information Sheet

University of North Carolina at Chapel Hill
Research Information Sheet
IRB Study #: 25-0459
Principal Investigator: Samantha Glover, DDS

With a prevalence of up to 11% of school-aged children and as high as 28% among adolescents, migraines are a significant concern in public health. Particularly as pediatric specialty providers, awareness of this particular ailment can be a key consideration for the way we may treat our patients. The main objective of this research study is to evaluate the level of education and training, assess clinical exposure to pediatric patients presenting with migraine-associated symptoms, identify potential gaps in training, and determine the level of interest among pediatric dentists and pediatric dental residents in expanding their role in pediatric migraine management to enhance interdisciplinary collaboration. We are specifically interested in understanding the knowledge, beliefs, patient identification, practice intervention, and future focus of pediatric migraine management.

Being in a research study is completely voluntary. You can choose not to be in this research study. You can also say yes now and change your mind later.

If you agree to take part in this research, you will be asked to evaluate your training and interest in pediatric migraine management and answer questions regarding patient population, education in migraine management, and orofacial comorbidities. Your participation in this study will take about 10-15 minutes. We expect that around 1000 people will take part in this research study.

You can choose not to answer any question you do not wish to answer. You can also choose to stop taking the survey at any time.

The possible risks to you in taking part in this research are:

- A minimal possible risk of loss of confidentiality if our collected data was to be compromised. Any information that is obtained in connection with this study and that you can be identified with, such as your email address, will remain confidential.

To protect your identity as a research subject, the research data will not be stored with your name or contact information, and the researchers will not share your information with anyone. In any publication about this research, your name will not be used. Additionally, there will be no analysis or mention of participating practices or institutions in reports that will emanate from this research project.

If you have any questions about this research, please contact Samantha Glover by calling 941-735-6937 or emailing sag40@ad.unc.edu. If you have questions or concerns about your rights as a research subject, you may contact the UNC Institutional Review Board at 919-966-3113 or by email to IRB_subjects@unc.edu.

○ I agree to take part in this research survey. I understand this survey is voluntary and I may choose not to participate at any time.

Import from library | Add new question

Add Block

Screening Question:

Q1 Are you currently completing or have already completed specialized training in pediatric dentistry f

Skip to

End of Survey if No Is Selected

Are you currently completing or have already completed specialized training in pediatric dentistry from a CODA-approved program in the United States?

- ◯ Yes
- ◯ No

Import from library | Add new question

Add Block

Professional Background:

Q2

What is your current professional role?

- ◯ Retired pediatric dentist
- ◯ Practicing pediatric dentist
- ◯ Pediatric dental resident
- ◯ Other (please list) __________

Q3

What is your primary practice setting?

- ◯ Private practice
- ◯ Academia
- ◯ Hybrid of private practice and academia
- ◯ Other (please list) __________

Q4

What type of location is your practice in?

- ◯ Urban
- ◯ Suburban
- ◯ Rural

Q5

Which region do you primarily practice in?

- ◯ Northeast
- ◯ Midwest
- ◯ West
- ◯ Southeast
- ◯ Southwest

Q6

How many years have you been in practice?

- ◯ 0-5 years
- ◯ 6-10 years
- ◯ 11-15 years
- ◯ 16-20 years
- ◯ Over 20 years

Q7 | Have you taken any additional training (certifications, continuing education, additional residency t

Have you taken any additional training (certifications, continuing education, additional residency training) in headache or migraine management?

- ◯ Yes (please specify)
- ◯ No

Import from library | Add new question

Add Block

General Understanding/Provider Knowledge

Q8 *

On a scale of 1-5 (with 1 being unaware and 5 being very aware):

	0	1	2	3	4	5
How aware are you of oral conditions that may contribute to migraines in pediatric patients? (i.e., including but not limited to temporomandibular disorders, bruxism, clenching)						

Q9 | What oral conditions have you seen associated with migraines in your pediatric patients? (choose all *

What oral conditions have you seen associated with migraines in your pediatric patients? (choose all that apply)

- ☐ Bruxism
- ☐ Temporomandibular disorders
- ☐ Clenching
- ☐ Attrition
- ☐ Malocclusion
- ☐ Other (please list) ____
- ☐ None

Q10 *

Did any of your educational background involve training on the role of pediatric dentists in migraine prevention or management?

- ○ Yes
- ○ No

Q11

Do you educate families on the connections between oral conditions and migraines in pediatric patients?

- Yes
- No

Page Break

Import from library | Add new question

Add Block

Patient Identification and Practice Interventions

Q12 | How often do your pediatric patients complain of headaches (including migraine) during consultations

How often do your pediatric patients complain of headaches (including migraine) during consultations?

- Frequently
- Occasionally
- Rarely
- Never

Q13 | On average, what percentage of your pediatric patients exhibit parafunctional habits like teeth grin

On average, what percentage of your pediatric patients exhibit parafunctional habits like teeth grinding or clenching (which can be linked to headaches or migraines)?

- Less than 10%
- 10-20%
- 20-50%
- 50-70%
- 70-90%
- 90-100%

Q14 | How frequently do your patients (or their parents) seek advice from you about headache or migraine m *

How frequently do your patients (or their parents) seek advice from you about headache or migraine management?

- ◯ Frequently
- ◯ Occasionally
- ◯ Rarely
- ◯ Never

Q15 | Are you equipped with resources (i.e., knowledge, training, collaboration with other specialists) to *

Are you equipped with resources (i.e., knowledge, training, collaboration with other specialists) to address the potential oral health-headache/migraine connection?

- ◯ Yes
- ◯ No

Q16 | 15. Do you believe more management is necessary with other healthcare providers (i.e. pediatricians, *

Do you believe more management is necessary with other healthcare providers (i.e. pediatricians, neurologists, orofacial pain specialists, nutritionists, etc.) to manage migraines in your pediatric patients?

- ◯ Yes
- ◯ No

Q17 | Do you coordinate care with other healthcare providers (i.e. pediatricians, neurologists, orofacial *

Do you coordinate care with other healthcare providers (i.e. pediatricians, neurologists, orofacial pain specialists, nutritionists, etc.) to manage migraines in your pediatric patients?

- ◯ Yes
- ◯ No

Q17b

Display this question

If Do you coordinate care with other healthcare providers (i.e. pediatricians, neurologists, orofacial Yes Is Selected

How often do you collaborate to manage migraines in pediatric patients?

- ○ Often (several times a year)
- ○ Rarely (a handful of times over the course of my career)
- ○ Never

Q18 In what ways have you recommended treatment for migraine management from the pediatric dentistry per

In what ways have you recommended treatment for migraine management from the pediatric dentistry perspective? (select all that apply)

- ☐ Occlusal guard (for nighttime wear)
- ☐ Occlusal splint
- ☐ Botulinum toxin type A
- ☐ Stress management modalities
- ☐ Orthodontics
- ☐ Nutritional guidance
- ☐ Referral to another dental specialist
- ☐ Referral to another medical specialist
- ☐ Working with a coordinated team of health care specialists
- ☐ Other (please specify)
- ☐ I do not recommend treatment

Import from library | Add new question

Add Block

Provider Opinion and Future Focus:

Q19

How much do you agree with the following statement:
It is important for pediatric dentists to understand the oral health- migraine relationship for their patients.

- ◯ Strongly disagree
- ◯ Somewhat disagree
- ◯ Neither agree nor disagree
- ◯ Somewhat agree
- ◯ Strongly agree

Q20

Do you believe further training or research in this area is necessary for pediatric dentists?

- ◯ Yes
- ◯ No

Q20b

Display this question

If Do you believe further training or research in this area is necessary for pediatric dentists? Yes Is Selected

What type of training would you recommend? (select all that apply)

- ☐ Continuing education courses focused on migraine management in pediatric dental patients
- ☐ Integration of migraine management education into pediatric dental residency programs
- ☐ Clinical rotations in residency focused on headache and migraine disorders
- ☐ More lectures at the AAPD Annual Session focused on the link between oral conditions and migraines
- ☐ Joint symposia/meeting of AAPD and the American Headache Society
- ☐ Other (please list)

Q21 What challenges have you had (or perceive to have) with implementing migraine management into your p *

What challenges have you had (or perceive to have) with implementing migraine management into your practice? (select all that apply)

- [ ] Lack of training or formal education in migraine management
- [ ] Difficulty distinguishing between dental pain and migraine symptoms
- [ ] Limited collaboration with other healthcare providers
- [ ] Time constraints
- [ ] Other (please list)

Q22 Which resources would help you integrate migraine management into your practice (select all that app *

Which resources would help you integrate migraine management into your practice (select all that apply)

- [ ] Evidence-based clinical guidelines from AAPD for migraine management in pediatric dental patients
- [ ] Access to referral networks for headache specialists or neurologists
- [ ] Resources on the relationship between oral conditions and migraines
- [ ] Tools for patient education on preventing and managing migraines
- [ ] Other (please specify)

Q23

Please share any other thoughts or feedback related to pediatric dentists' management of migraines.

Import from library

Add new question

Add Block

Demographics

Q24 *

What is your age?

- ○ Under 25
- ○ 25-29
- ○ 30-39
- ○ 40-49
- ○ 50-59
- ○ 60 or older
- ○ Prefer not to answer

Q25 *

What gender do you identify with?

- ○ Male
- ○ Female
- ○ Non-binary / third gender
- ○ Prefer not to say

Q26 *

Which ethnicity do you identify with?

- ○ Hispanic or Latinx
- ○ Not Hispanic or Latinx
- ○ Unsure
- ○ Prefer not to answer

Q35

*

What race do you identify with?

- White
- Black or African American
- American Indian or Alaska Native
- Asian
- Native Hawaiian or Other Pacific Islander
- Other
- Unsure
- Prefer not to answer

Import from library

Add new question

Add Block

End of Survey

We thank you for your time spent taking this survey.

Your response has been recorded.
